# Tobacco use among male inmates and their attitudes toward its prevention in Khartoum State: A cross-sectional study

**DOI:** 10.18332/tpc/109784

**Published:** 2019-07-19

**Authors:** Elhadi M. Awooda, Dina E. Shashati

**Affiliations:** 1 Department of Restorative Dentistry, University of Medical Science and Technology, Khartoum, Sudan

**Keywords:** incarceration, male prisoners, tobacco use, toombak, tobacco cessation

## Abstract

**INTRODUCTION:**

Tobacco use remains the most preventable cause of death and disability, with prisoners being a neglected population of tobacco users. The aim was to determine the prevalence of tobacco use and attitudes toward its prevention among adult male prisoners.

**METHODS:**

We conducted a descriptive cross-sectional study among 349 adult male inmates from three prisons in Khartoum State. The interview questionnaire included questions related to tobacco use status, type of tobacco used, previous attempts to quit, smoking inside a room, frequency of tobacco use before and after incarceration, and attitude toward its prevention. Chi-squared and paired t-test were used to compare between different variables, with the level of significance set at p≤0.05.

**RESULTS:**

The majority (69.1%) were in the age group 30–50 years. All of the studied prisoners were tobacco users of which: 43.8% used oral snuff (toombak); 22.1% were cigarette smokers; 30.9% used both cigarette and toombak; 0.6% used cigarette and waterpipe; and 3.2% used cigarette, toombak and waterpipe. Toombak users (alone or with other types of tobacco) were 272 (78%) with the majority (62.4%) dipping 2–5 times per day. There were 96 (57%) cigarette smokers (alone or with other types), and waterpipe users were 12 (3.8%). The majority (74.6%) of cigarette users shared their cell with other toombak users. For the majority (79.6%), the number of cigarettes and snuff dipping per day was directly proportional to the period of incarceration. Almost all (99.1%) prisoners know the harmful effects of tobacco use, and 64.5% had previously attempted to quit. Also, 98% of tobacco users reported a desire to quit and expressed willingness to participate in a tobacco-cessation program.

**CONCLUSIONS:**

Different patterns and methods of tobacco use were explored, and all the studied prisoners were users. Tobacco use increased after incarceration. The willingness to participate in tobacco-cessation counselling should be met with the implementation of a planned and well-designed prevention program.

## INTRODUCTION

Tobacco use is one of the biggest health concerns in the world today^1^, killing nearly half of its users^2^. According to WHO 2017, there are no data on the prevalence of tobacco use in Sudan^3^. However, in Sudan, there is known consumption of smokeless tobacco (snuff) locally named toombak^4^. Toombak is a very popular substance used by the Sudanese community and represents an integral part of their culture^5,6^. It is not chewed but rather dipped and retained between the gums and the lips, cheeks, or floor of the mouth, and sucked slowly for approximately 10–15 min^7^. The prevalence of toombak use is around 47% among men aged 40 years and older and 10% among women aged 60 years and older^8^. Oral cancer in Sudan is ranked as sixth amongst all cancer types (6.1 per 100000)^9^. Toombak is known to play a significant role in the etiology of oral cancer^10^ and is a major risk factor for oral squamous cell carcinoma^11^. The negative health consequences of smokeless tobacco use and its relatively high prevalence make it necessary to promote its cessation^10^.

Smoked tobacco use in Sudan includes cigarette and waterpipe (locally named shisha). Cigarette smoking has a prevalence of 20% among Sudanese males in the young population^12^. Waterpipe (WP) is a traditional device used for tobacco smoking attached to a water bowl^13^. WP use has a history of about 400 years with the device having different names like: shisha, narghile, hookah, chillum, and arghile^13-15^. It is considered as a causative factor for many diseases^16^.

Tobacco has long been considered part of prison culture and the smoking situation among prisoners is more severe^17^. Studies have shown a high prevalence of smoking within correctional facilities^18^. Many efforts have been made to help the general population to cease tobacco use, but on the other hand, this problem among prison populations is neglected^19^. However, incarceration might provide an opportunity to promote tobacco-use cessation.

A 2015 literature review identified just 12 papers examining smoking cessation in the male prisoner population^20^. Most of the studies focus on smoking prevalence and/or the efficacy of behavioral or pharmacological interventions. The case may be the same in Sudan, and it can be hypothesized that tobacco prevalence is high among Sudanese prisoners, but qualitatively their tobacco-use types, attitudes towards tobacco cessation, motivation to quit, and awareness of the harmful effects of tobacco on health have not been investigated. Despite evidence of high rates of tobacco use among male prisoners worldwide and the danger of passive smoking, there has been no research that describes tobacco use patterns and associated factors among this group of people in Sudan.

The general objective of this study was to assess the prevalence of tobacco use among a sample of male prisoners in Khartoum State. The specific objectives were to compare prisoner attitudes towards tobacco use before and after incarceration; to assess the relationship between the period of incarceration and frequency of tobacco use and their willingness to quit; and to assess descriptors of tobacco use (number of cigarettes or snuff dipping used and years of use) in relation to incarceration.

## METHODS

A descriptive cross-sectional study was conducted among male prisoners from three prisons in Khartoum State, Sudan. The total number of prisoners was 3800, the sample size was calculated by anticipating at least 20% prevalence of tobacco smoking from recent study results conducted among Sudanese adult youth^15^ with z=1.96 for a 95% confidence level, margin of error of 5%, and expected response rate of 50%. The minimum sample size required to estimate population parameters was estimated as 349. The studied populations were selected on the basis of inclusion criteria: aged >18 years, male gender, Sudanese nationality, and those who have spent more than 1 year inside the prison. Three male prisons were selected at random, based on different geographical locations in the capital Khartoum State. The sample size was divided proportionally according to the total number from each of the three prisons. Then, from each prison, systematic random sampling was used to select the participants based on their incarcerated numbers. Names and numbers of prisoners were kept anonymous for confidentiality.

The interview questionnaire included closed-ended questions on: sociodemographic data, tobacco use, types of tobacco use, frequency of tobacco use before and after incarceration, sharing cells with non-smokers, smoking inside cells, willingness to quit tobacco use, and previous attempts to quit. The questionnaire was piloted among 20 volunteer prisoners in order to establish its validity and comprehensibility, with a Cronbach’s alpha coefficient of 0.87.

The study was approved by the Ethical Committee of the University of Medical Science and Technology. Permission to conduct the study in the prisons was signed by the Directors of Prisons and Reform (State of Khartoum) after the approval of the Director of the Department of Health and Medical Services of Khartoum Prisons. Before the completion of the questionnaire, the eligible participants were informed about the aim of the study and requested to participate voluntarily, those who accepted signed an informed written consent. The participants were free to leave any question unanswered for any reason whatsoever. The questionnaire was administered in the form of extensive face-to-face interviews for both literate and illiterate prisoners, as one previous study indicated that the majority of prisoners were illiterate^21^. The interview was carried out in an office provided by the prison authorities to ensure the privacy and confidentiality of the participants. The participants were interviewed by one trained interviewer (the second author).

Data were analyzed using SPSS software (version 21; SPSS Inc., Chicago, IL). Comparison between non-parametric data was done using a chi-squared test. In the case of parametric data, a paired t-test was used to examine changes in the number of cigarettes smoked and toombak dipped, measured in average cigarettes smoked or toombak dipped per day. A Wilcoxon signed rank test was used to assess changes in the frequency of toombak, cigarettes or shisha following incarceration. The level of statistical significance difference was set at p≤0.05.

## RESULTS

A total of 349 prisoners completed the questionnaire. The majority of the sampled prisoners (69.1%) were in the age group 30–50 years, and 82% had spent more than 5 years in prison. The prevalence of tobacco use was extremely high: all respondents (100%) were users; 43.8% reported using toombak; 22.1% were cigarette smokers; 30.9% were both cigarette and toombak users; 0.6% cigarette and waterpipe users; and 3.2% cigarette, toombak and waterpipe users.

Any toombak use (alone or with other types of tobacco) was noted in 272 respondents (78%) with the majority (62.4%) dipping 2–5 times per day. There were 96 (57%) cigarette smokers (alone or with other types). The association between the frequency of cigarette smoking and toombak use with the period of incarceration is shown in Tables 1 and 2, respectively. Waterpipe users were 12 (3.8%), while the frequency of use per week was: 41.7% three times or more, 50% twice or less, and 8.3% once.

**Table 1:** Association between the frequency of cigarette smoking and the period of incarceration among Sudanese prison inmates

*Period of incarceration*			
*Number of cigarettes per day*	*One to 5 Years n (%)*	*More than 5 years n (%)*	*Total*
<10	55 (39.6)	11 (19.3)	66 (33.7)
10–20	69 (49.6)	32 (56.1)	101 (51.5)
>20	15 (10.8)	14 (24.6)	29 (14.8)
Total	139 (100)	57 (100)	196 (100)

p=0.014

**Table 2:** The association between the frequency of toombak dipping per day and the period of incarceration in Sudanese prison inmates

*Period of incarceration*			
*Frequency of toombak dipping per day*	*One to 5 years n (%)*	*More than 5 years n (%)*	*Total*
1	19 (90.5)	2 (9.5)	21 (100)
2–5	139 (81.3)	32 (18.7)	171 (100)
>5	58 (70.7)	24 (29.3)	82 (100)
Total	216 (78.8)	58 (21.2)	274 (100)

The majority of the 160 (81.6%) smokers and of the 207 (75.5%) toombak users started tobacco use before incarceration, while 36 (18.4%) of current smokers and 67 (24.5%) of current toombak dippers used tobacco after incarceration. The majority of smokers (147, 74.6%) shared their cell with toombak users, and almost more than half (110, 55.8%) smoked inside their cells.

Almost all (99.1%) prisoners knew about the harmful effects and malignant diseases associated with tobacco use. The majority (64.5%) of smokers had previously attempted to quit, and 98% of them were willing to participate in tobacco cessation programs.

## DISCUSSION

Prisoners in this study were willing and motivated to participate. Level of education, age and marital status were not considered as variables in this study as the main objectives were to determine the prevalence of tobacco use in relation to incarceration period and their attitude toward tobacco cessation. Also, years of incarceration (the duration since the beginning of incarceration) was limited to one year (as a minimum) as we considered that less than a year in prison might be the reason a prisoner used tobacco to cope with the shorter duration, so this should be considered when comparing with more than a 5-year incarceration.

Additionally, the sample size of 349 was calculated from the prevalence of cigarette smoking of 20%^12^, and not from the toombak prevalence of 47% among the Sudanese adult population^8^, since before we conducted the study we tried to eliminate a type II error, but the result was compromised by a type I error, as the prevalence of tobacco use was 100%.

The prevalence of tobacco use among Sudanese prisoners was higher than the highest percentages among other countries worldwide^17-19,22,23^, which may be attributed to the use of toombak, which easily finds its way into prisons. It does not need to be burnt and can be used inside the cell without drawing the attention of prison authorities. As this is the first study to determine the prevalence of tobacco use among prisoners in Sudan, the high prevalence of current toombak users (78%) should stimulate studies of this phenomenon and of the preventive and cessation methods needed to be implemented. Also, this high prevalence necessitates a re-evaluation of old data that stated that 47% of the Sudanese adult male population are toombak users^8^. Unfortunately, in many countries, cessation programs and tobacco bans, in general, are focused on smoking and ignore other types of tobacco use including smokeless tobacco and waterpipe.

In recent years, there has been an alarming increase in the global prevalence of waterpipe use, particularly among the youth^15,24^. One main reason for this popularity is the general belief that waterpipe is safe because the smoke passes through water, and its harmful effects are ‘filtered’^25^. Our results reveal that waterpipe was the least common type of tobacco used with a percentage of 0.6%, which is very low compared to the results for non-prisoner male university students from some other countries^16,24,26,27^. Waterpipe has been recently banned by the Khartoum State Ministry of Health Authority in public areas, including prisons. This may justify the very low percentage of its use in the prisons.

As the majority of prisoners started tobacco use before incarceration, this may be a reflection of prisoner personality types, or may indicate the high prevalence of tobacco use among the Sudanese population. However, this hypothesis needs to be investigated. An increase in the frequency of tobacco use was observed among those prisoners who started tobacco use before imprisonment, which is similar to that observed by Kauffman et al.^28^. Tobacco use increased after incarceration because prisoners were more likely to be exposed to negative health behaviors, which they may then adopt as a means of coping with the stresses of prison life and being away from family and home. Toombak use may also be considered in psychological terms to facilitate social networking between prisoners and a bag containing almost 300 g of snuff can be shared conscientiously with a large number of prisoners, especially those who do not have parents or relatives as visitors. Also, it is used as a substitute for cigarette smoking to satisfy nicotine addiction. Unfortunately, there are no educational, counselling, or preventive measures regarding the harmful effects of toombak, neither in the community nor in the prisons.

The high percentage of prisoners who smoke inside their cells shared with a non-smoker (but toombak users) pose a risk of secondhand smoking. This causes severe health problems and a threat for non-smoking prisoners and even prison staff. As smoker prisoners may share their cells with non-smokers, this has led, in some countries, to the banning of tobacco use inside cells or corridors^17,28,29^.

In agreement with the results of the study by Kauffman et al.^28^, the increase in tobacco use among prisoners who spent more than 5 years in prison compared with prisoners having a shorter sentence of 1 to 5 years may be because those prisoners have failed to cope with being a prisoner and with the stress associated with incarceration or peer pressure.

Similar to other studies^18,28^, almost all prisoners were aware of the harmful effects and malignant diseases associated with tobacco use. Additionally, our results are concomitant to those of Tiwari et al.^30^ where the majority of prisoners had previously attempted to quit, but failed to achieve this goal. Prisoners want to quit and change their lifestyle^28,31^, but they cannot do it without help. Besides tobacco cessation counselling, current guidelines recommend the use of nicotine replacement products or medications to assist with tobacco cessation^32^. In contrast, a recent study in the Northern Territory of Australia by Hefler et al.^33^ concluded that many prisoners expressed a preference for cessation support options other than nicotine replacement therapy (NRT).

The majority of prisoners were willing to participate in a tobacco-cessation program, which should be tailored to the unique stress of the prison environment and implemented to help willing prisoners. The legal obligation to implement strategies for smoke-free environments, including prisons, is mandated by the World Health Organization Framework Convention on Tobacco Control^34^. In some developed countries, tobacco smoking has been fully or partially banned in correctional facilities^1,17,22,35,36^, which could be useful for all smokers, non-smokers, and prison staff^37^, but still some concluded that forced abstinence (a total or partial smoking ban) had little impact on tobacco cessation among prisoners^20^. There is also some evidence that bans can have unintended consequences including aggressive behaviour^35^ and future relapse to smoking following release from smoke-free correctional facilities^38^.

Furthermore, some researchers believe that tobacco users must quit by themselves^30^. Efforts and interventions to help prisoners to cease tobacco use show more significant improvement among prisoners than from merely preventing them from using tobacco while incarcerated^33,39^. In the Sudan context, we think efforts to reduce cigarette tobacco use in prisons need to consider the specific factors influencing smoking habits in prisons and any unbalanced attempts at reducing cigarette smoking may create a shift to toombak.

### Limitations

While novel, this study is not without limitations. This descriptive cross-sectional study makes no claim of generalizability and cannot be taken to represent all prisoners. Females and youths were not represented in the sample. Also, age, marital status, job/profession and educational level were not considered variables as it is the first study to explore the prevalence of tobacco use in Sudanese prisons, the type and associated factors like years of incarceration, association with the frequency of tobacco use, quitting and attitude towards its prevention.

## CONCLUSIONS

The prevalence of smoked and smokeless tobacco (toombak) use among Sudanese prisoners is high. Incarceration had no association with the initiation of tobacco use but had a major role in the increase in the frequency of use. Frequency of smoking and toombak dipping increased with an increase in the period of incarceration. Almost all prisoners in the study knew the harmful effects of tobacco use and expressed willingness to participate in tobacco-cessation programs.
